# Rapid and robust isolation of microglia and vascular cells from brain subregions for integrative single-cell analyses

**DOI:** 10.1016/j.heliyon.2024.e35838

**Published:** 2024-08-05

**Authors:** Efthalia Preka, Alejandro Lastra Romero, Ying Sun, Yara Onetti Vilalta, Thea Seitz, Adamantia Fragkopoulou, Christer Betsholtz, Ahmed M. Osman, Klas Blomgren

**Affiliations:** a Department of Women's and Children's Health, Biomedicum A4, Karolinska Institutet, 171 77, Stockholm, Sweden; b Department of Immunology, Genetics and Pathology, Rudbeck Laboratory, Uppsala University, Sweden; c Department of Medicine Huddinge, Campus Flemingsberg, Neo, 141 57, Huddinge, Sweden; d Pediatric Oncology, Karolinska University Hospital, 171 64, Stockholm, Sweden

**Keywords:** Neuroinflammation, Vascular disease, Endothelial cells, Pericytes, RNA sequencing

## Abstract

Cell isolation protocols from brain tissue include prolonged *ex vivo* processing durations, rendering them suboptimal for transcriptomic studies. Particularly for microglia and vascular cells, current isolation methods produce lower yields, necessitating addition of an enrichment step, and use of large tissue volumes - in most cases whole brain tissue - to obtain sufficient yields. Here, we developed a simple, rapid, and reproducible cell isolation method for generating single-cell suspensions from micro-dissected brain regions, enriched for microglia and vascular cells, without an enrichment step. Cells isolated using this method are suitable for molecular profiling studies using 10 × Genomics Chromium single-cell RNA sequencing with high reproducibility. Our method is valuable for longitudinal unbiased molecular profiling of microglia and vascular cells within different brain regions, spanning multiple time points across physiological development or disease progression.

## Motivation

Physiological development and disease progression are dynamic processes. In the brain, microglia and vascular cells undergo frequent remodeling, and yet there are no longitudinal studies based on unbiased molecular profiling of these cell types at a single-cell level, especially within a specific brain region. This is largely due to a lack of reproducible cell isolation methods generating robust yields of viable cells from micro-dissected brain regions. Current cell isolation protocols are cumbersome and use whole cerebral tissue, followed by an enrichment step, to obtain sufficient cellular yields. This approach is disadvantageous, as it may mask the cellular heterogeneity within a specific brain region. Moreover, the selection process is based on classical markers, potentially hindering the discovery of novel subtypes. Here, we describe a simple, rapid, and reproducible cell isolation method for generating single-cell suspensions from micro-dissected brain regions that favors the capture of microglia and vascular cells without an enrichment step, and which is suitable for integrative longitudinal molecular studies using single-cell RNA sequencing.

## 1 Introduction

Single-cell transcriptomic analyses have advanced the understanding of the cellular compositions and molecular states in multiple organs under homeostasis and pathology, including those of the central nervous system (CNS) [1]. Despite this technological breakthrough in biology, the utility of this technique in studies performed in solid organs is often compromised by cumbersome cell isolation methods. Multi-step cell isolation protocols prolong *ex vivo* tissue handling, that may result in inaccurate molecular profiles and yield lower numbers of viable cells, especially in sensitive organs such as the brain. Current CNS tissue dissociation protocols apply longer incubation in enzymatic solutions combined with frequent application of mechanical force to achieve a complete dissociation [2]. Especially for CNS tissue, lengthy cell isolation protocols are often unavoidable due to inclusion of the essential, but laborious, myelin removal step [[2], [3], [4], [5]]. Myelin removal is critical for successful downstream cell applications, such as cell sorting or processing for single-cell RNA sequencing (scRNA-seq) using a droplet-based method, such as 10 × Genomics Chromium. The combination of longer processing durations, harsh tissue handling in enzymatic solutions, and the prolonged myelin removal steps, lead to lower cellular yields. These factors are critical issues for sensitive cell types, such as microglia and vascular cells. To acquire a better yield of these sensitive cells, current cell isolation protocols require use of more tissue, whole cerebral tissue in most cases, followed by an enrichment step using antibodies or genetically labeled cells with fluorescent reporters under certain promoters [2,3,6]. However, this approach has several disadvantages: a) it hinders studying the cellular heterogeneity within a specific brain region, and b) the enrichment process increases *ex vivo* cell handling and reduces the power of the single-cell analysis in discovering unique subtypes associated with certain developmental or disease states, as the selection is based on classical predefined markers. Here, we established a simple, rapid, and reproducible cell isolation method for generation of single-cell suspensions from micro-dissected brain regions, favoring the capture of microglia and vascular cells, without a preselection step, that is suitable for longitudinal molecular profiling studies using scRNA-seq.

## 2 Materials and methods

### 2.1 Animals

All experimental procedures were carried out according to the European and Swedish animal welfare regulations and were approved by the Northern Stockholm Ethical Committee (application nr. N141/16). Female C57Bl/6J (Charles River, Sulzfeld, Germany, stock #000664) were used. After delivery from the vendor, animals were allowed to acclimate for at least one week in the following housing conditions: equal light/dark cycle (12/12 h), 20–22 °C ambient temperature and ∼80 % relative humidity, with access to food and water *ad libitum*. For the cerebral cortex, cells were isolated from three-week-old and nine-week-old mice. For the hippocampus, cells were isolated from five-week-old mice. Cells were isolated from three mice per each cortical or hippocampal preparation.

### 2.2 Tissue collection and generation of the single-cell suspension

Animals were deeply anesthetized with sodium pentobarbital (100 mg/kg, ABCUR AB #444362, Sweden) and transcardially perfused with 20 ml of ice-cold 1 × phosphate buffered saline without Ca^2+^ and Mg^2+^ (PBS; pH 7.4; Gibco/Life Technologies #10010056), using a 20 ml syringe equipped with a 23-gauge blunt needle. The animal head was detached and sprayed with 70 % ethanol. The brain was removed from the cranium and placed onto an inverted Petri dish. Cerebral cortices and hippocampi of both hemispheres were dissected at room temperature using 45°-angled fine forceps. Every effort was made to remove the white matter remaining attached to the region of interest from the adjacent corpus callosum by a applying gentle unidirectional scraping using a 45°-angled fine forceps. Dissected tissues were placed into 1.5 ml microtube containing ice-cold 1 × PBS, then placed on ice until all dissections were completed. E. All downstream cell isolation steps were performed in a sterile laminar flow hood using sterile ice-cold solutions, except for the enzymatic digestion step that required an incubation at 37 °C (detailed below). The microtubes containing the dissected tissues were wiped off with 70 % ethanol and the PBS was removed. Tissues were washed once with 1 ml of 1 × Hanks's balanced salt solution without phenol red, Ca^2+^ and Mg^2+^ (HBSS; Gibco/Life Technologies #14175095). Dissected tissues were placed onto a glass slide, and the remaining white matter pieces were further removed by a gentle unidirectional scraping using two pipette tips, one for stabilizing the tissue and the other for scraping. The tissue was chopped into small pieces using a scalpel (10–15 strokes in multiple directions) and transferred into a 15 ml conical tube containing 1 ml of an enzymatic mixture of dispase II (1 mg/ml; Sigma-Aldrich #D4693), papain (0.1 mg/ml; Roche #000000010108014001) and DNase I (0.5 mg/ml; Roche # 000000010104159001) made in 1 × HBSS containing 12.4 mM magnesium sulfate (Sigma-Aldrich #M7506) as an enzyme cofactor [[7], [8], [9]]. Tubes were incubated at 37 °C for 10 min without agitation. The enzymatic activity was reduced by adding 200 μl ice-cold heat inactivated fetal bovine serum (FBS; Gibco/Life Technologies #10500064) and the tissues were homogenized by gentle pipetting up and down (5–10 times) until no tissue pieces were visible. The cell suspension was filtered through a 70-μm cell strainer (Miltenyi Biotec #130095823) and placed into a new 15 ml conical tube. The enzymic activity was stopped by adding 4 ml of 20 % ice-cold FBS solution made in 1 × HBSS. The cell suspension was centrifuged at 500×*g* at 4 °C for 5 min using an Eppendorf 5702R centrifuge. For myelin removal, the cell pellet was resuspended in 1 ml of 20 % Percoll® solution (Percoll® plus, GE Healthcare, #GE17-5445-01) made using 10 × phenol red HBSS (Gibco/Life Technologies #14060040) and 1 × HBSS. After complete resuspension of the cell pellet, an additional 4 ml of the 20 % Percoll® solution was added, bringing the volume up to 5 ml. The suspension was then gently overlaid with 5 ml of 1 × HBSS. Tubes were spun at 500×*g* at 4 °C for 10 min with no break. The supernatant containing the myelin debris was carefully discarded, and the cell pellet was resuspended in 1 ml flow cytometry buffer (R&D Systems #FC001). At this step, samples from the three animals were pooled into one 15 ml conical tube and centrifuged at 500×*g* at 4 °C for 5 min. The supernatant was carefully discarded, and the cell pellet was resuspended in 500 μl flow cytometry buffer. The cell suspension was transferred into a 1.5 ml microtube. Cell viability was accessed using the trypan blue exclusion method and processed for single-cell RNA sequencing.

### 2.3 Single-cell RNA sequencing

Isolated cells were processed for single-cell RNA sequencing at the eukaryotic single-cell genomics facility (ESCG) at Karolinska Institutet, Sweden using the 10 × Genomics Chromium method (version 3.0.1) and sequenced using Illumina NovaSeq 6000 with an S2 flow cell. In each preparation, we aimed to sequence 15 × 10^3^ cells processed in triplicates for scRNA-seq, *i.e.* 5 × 10^3^ per replicate, to minimize the number of doublets, previously shown to be proportional to the cellular input (loaded cells) [10,11].

The Cellranger 5.0.1 pipeline was used to align the raw sequencing reads to the mouse reference genome, *Mus musculus* version *mm10,* and generate the unique molecular identifier (UMI) count matrix. The generated count matrix was analyzed using the Seurat R package (version 3.0.0). Each preparation included 3 technical replicates and each replicate was processed independently before integrating them together. Low-quality cells were excluded based on the number of genes expressed in the cells and the percentage of mitochondrial counts. Cells expressing between 250 and 6000 genes were considered for downstream analysis, given that each gene was expressed in at least three cells.

The mitochondrial counts were set at 25 % and 15 % when analyzing all cell types and vascular cells, respectively. The filtered count matrices were normalized by dividing the feature counts for each cell by the total number of counts from all cells, then multiplied by 10,000 and log-transformed. Next, we identified highly variable features for each dataset independently. We used the default Seurat method for the integration of datasets and removal of the batch effect. Briefly, we used canonical correlation analysis and identified “anchors” (conserved cell groups) between datasets, which were used for batch correction and for the comparison of differentially expressed genes between different experimental conditions. The data were scaled using Pearson residuals, regressing out the variation by the mitochondrial genes. Principal component analysis (PCA) was then performed on the scaled data for dimensionality reduction, using 30 components. The output was used to produce a uniform manifold approximation and projection for dimension reduction (UMAP) with a resolution of 0.5 for identification of the clusters.

Identification of each cluster was based on signature genes of each cell type according to the existing literature as well as the automatic cell type annotation package SingleR. Signature genes for microglia included *Sall1*, *Cx3cr1*, *Tmem119*, *P2ry12*; for macrophages *Mrc1* and *Ms4a7*; for monocytes *Ly6c2, Cytip* and *Lyz2*; for oligodendrocytes, *Pdgfra*, *Olig2* and *Mog*; for astrocytes *Gja1, Gfap* and *Aqp4*; for neurons and neuronal progenitors *Dcx*, *Rbfox3* and *Syt1*, and *Gad1*; for endothelial cells *Cldn5*; for pericytes *Kcnj8* and *Pdgfrb*; for fibroblasts/myofibroblasts *Col1a1* and *Acta2*; for B cells *Cd79a, Cd19 and Igkc*; for T- and natural killer cells *Cd7, Cd3g* and *Itgb7.*

## 3 Results

### 3.1 Rapid generation of single-cell suspensions from a micro-dissected brain region

**Fig. 1:**
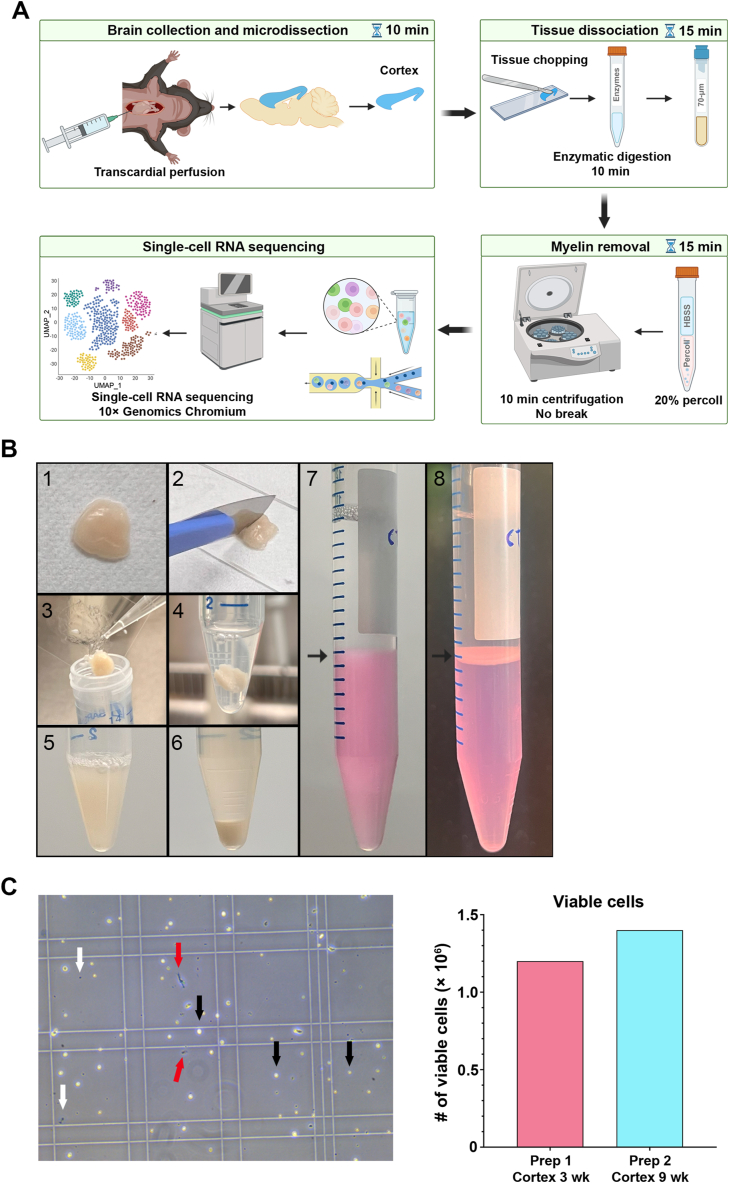
Rapid isolation of viable cells from micro-dissected brain regions (A) Schematic illustrating the steps of the single-cell isolation protocol. (B) Representative images displaying the cortical tissue handling through the different steps of generation of the single-cell suspension, starting by placing the harvested tissue onto a glass slide [1], followed by tissue chopping [2] and transferring the chopped tissue into a 15 ml conical tube [3] containing 1 ml of the enzymatic solution [4]. After incubation for 10 min, followed by several gentle pipetting up and down, the tissue is fully digested, producing a homogenous solution, allowing filtering the obtained cell suspension into a new conical tube [5]. The suspension is then spun to pellet the cells and myelin debri in the bottom of the tube [6]. The pellet is resuspended in the Percoll solution (7; bottom pink solution) and overlaid with HBSS (top clear solution). The black arrows indicate the interface between the two solutions, before the centrifugation [7], and after, where the myelin debri accumulate [8] and the cells remain in the bottom of the tube. (C) Viability of isolated cells. Left: Representative image of trypan blue exclusion for assessing cell viability. Black arrows indicate viable cells; white arrows indicate dead cells; red arrows indicate debris. Right: Bar plot showing the number of viable cells obtained from two independent preparations from the cerebral cortex of three- or nine-week-old mice, Prep 1 and Prep 2, respectively. (For interpretation of the references to colour in this figure legend, the reader is referred to the Web version of this article.)

We harvested cerebral cortices on two independent occasions (6 weeks apart) from mice of different ages. In preparation 1 (prep 1) mice were three-week-old (juvenile), and in prep 2 mice were nine-week-old (young adult). The time required per animal for anesthesia, perfusion and micro-dissection was 10 min when these processes were handled by two individuals. In this study, cerebral cortices were collected from three mice per age group, thus the required time for collection of cortices was limited to 30 min. We optimized a method for complete tissue dissociation with a short incubation step of 10 min at 37 °C, without agitation, in an enzymatic solution containing dispase II (1 mg/ml), papain (0.1 mg/ml) and DNase I (0.5 mg/ml). We also optimized this method for rapid removal of the myelin debris using a single gradient of Percoll® solution (20 %) that required only 10 min centrifugation. Samples from individual animals per preparation were handled independently to achieve an efficient dissociation and myelin removal, after which the obtained cells from each sample were pooled. These independent cortical preparations yielded comparable numbers of viable cells (prep1: 1.2 × 10^6^; prep 2: 1.4 × 10^6^) and were directly processed for scRNA-seq using a droplet-based method (10 × Genomics Chromium), without any additional purification or enrichment step (Fig. 1A–C).

### 3.2 Consistent cell isolation method appropriate for investigating microglial and vascular transcriptomics

**Fig. 2:**
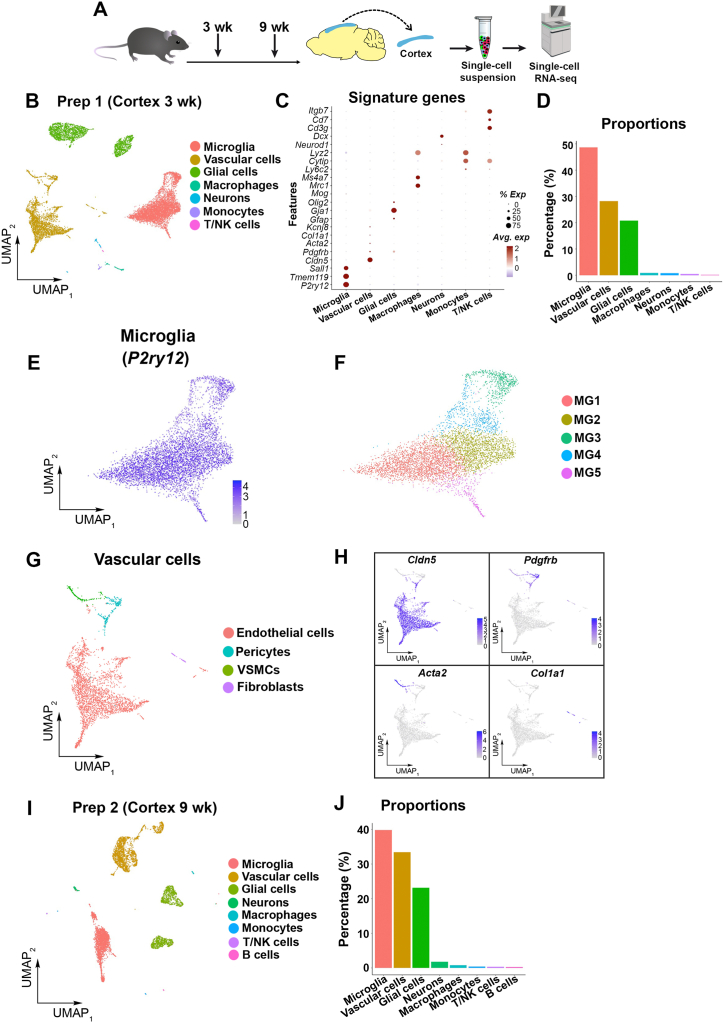
**Consistent cell isolation method appropriate for investigating of microglial and vascular transcriptomics.** Also see Supplementary Fig. 1. (A) Scheme illustrating the experimental design. wk = weeks. 3 wk, n = 3; 9 wk, n = 3. (B) Uniform manifold approximation and projection (UMAP) showing clustering of cell types obtained from the cerebral cortex of three-week-old mice (Prep 1). NPCs = Neural progenitor cells. T/NK = T- and natural killer cells. (C) Dot plot showing the expression of signature genes identifying the captured cell populations. (D) Bar plot showing the proportions of different cell types obtained from the cerebral cortex of three-week-old mice. (E) UMAP visualizing the microglial population obtained from the cerebral cortex of three-week-old mice, identified by *P2ry12* expression. (F) Subclustering of microglial cells revealed five unique subtypes (MG1 - MG5). (G) UMAPs visualizing the vascular cell types obtained from the cerebral cortex of three-week-old mice, endothelial cells (*Cldn5*), pericytes (*Pdgfrb*), vascular smooth muscle cells (VSMCs; *Acta2*), and fibroblasts (*Col1a1*). (H) UMAP showing clustering of cell types obtained from the cerebral cortex of nine-week-old mice (Prep 2). (I) Bar plot showing the proportions of different cell types obtained from the cerebral cortex of nine-week-old mice.

Cell suspensions generated from the abovementioned two independent preparations, were processed for scRNA-seq (Fig. 2A). In prep 1 (from three-week-old mice), after performing the quality control on the sequenced cells, we obtained ∼13 × 10^3^ cells. Phenotypic signature genes revealed that these cells were microglia, vascular cells, glial cells (astrocytes, radial glia and oligodendrocytes), neurons and neural progenitor cells (included in the neuronal cluster), and immune cells, these cell types were represented in all sequenced triplicates (Fig. 2B and C; Supplementary Figs. 1A and 1B). Of these cell types, microglia were the most abundant cells (∼50 %), followed by vascular cell (endothelial cells, and mural cells; ∼25 %), and glial cells (∼20 %) (Fig. 2D). We decided to analyze the microglia and vascular cells in depth as they were the most abundant cell types, and are rarely captured by currently available isolation protocols unless an enrichment step is applied [6], especially from a micro-dissected brain region. Subclustering of microglia revealed five unique clusters (Fig. 2E and F; Supplementary Fig. 1C), indicating that our isolation method allows capturing and analyzing microglial subtypes. Subclustering of vascular cells revealed the following populations: endothelial cells (*Cldn5*), pericytes (*Pdgfrb*), vascular smooth muscle cells (VSMCs; *Acta2*) and fibroblasts (*Col1a1*) (Fig. 2G). Within the endothelial cells, we were able to detect cells composing different vascular structures, such as arterioles (*Vegfc*), venous (*Nr2f2*), and capillaries and venules (*Slc16a1*) (Supplementary Fig. 1D). In prep 2 (from nine-week-old mice), after the quality control evaluation, we obtained ∼6.5 × 10^3^ cells, and after applying the abovementioned cell analyses, we were able to reproduce similar findings regarding the identity of the obtained cell types and their abundancy (Fig. 2H and I; Supplementary Fig. 1E).

Collectively, our cell isolation method produces a cell suspension suitable for performing successful and reproducible scRNA-seq using the droplet-based method, and robustly captures microglia and vascular cell subtypes, without a preselection step. This protocol could thus be leveraged for execution of across-time molecular kinetic studies within specific brain regions on aforementioned cell types under physiological development or a pathological condition.

### 3.3 Reproducible cell isolation and scRNA-seq data across brain regions

**Fig. 3:**
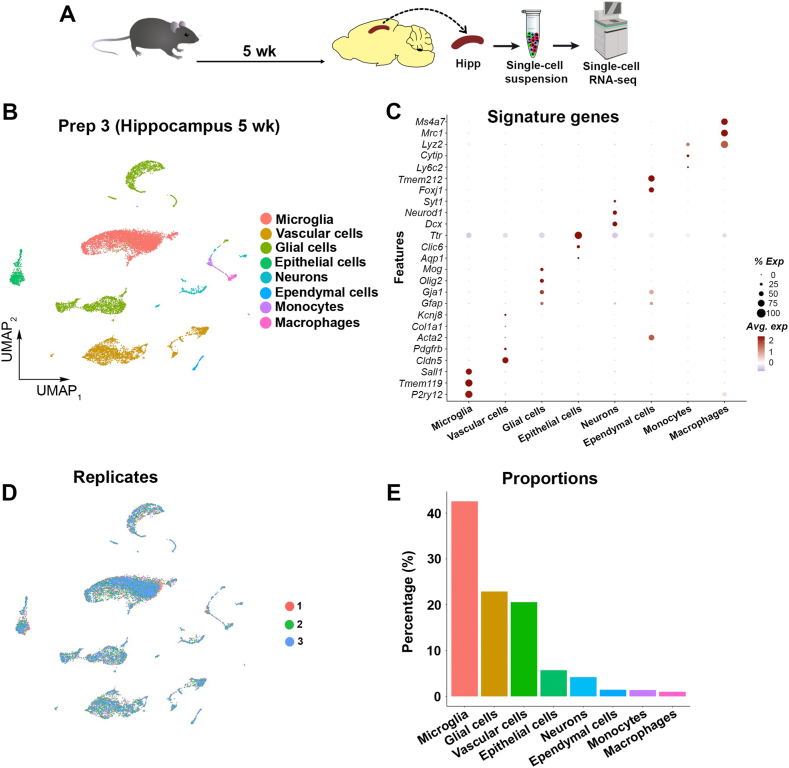
Reproducible cell isolation and scRNA-seq data across brain regions (A) Scheme illustrating the experimental design. n = 3. (B) UMAP showing clustering of cell types obtained from the hippocampus of five-week-old mice (Prep 3). (C) Dot plot showing the expression of signature genes identifying the captured cell populations. (D) UMAP displaying cells obtained from the three sequencing replicates performed in Prep 3 (replicate 1 = red, replicate 2 = green, replicate 3 = blue). (E) Bar plot showing the proportions of different cell types obtained from the hippocampus of five-week-old mice. (For interpretation of the references to colour in this figure legend, the reader is referred to the Web version of this article.)

We next wanted to challenge the reproducibility and robustness of our cell isolation method in relation to microglia and vascular cell enrichment by performing a third isolation experiment (preparation 3; prep 3), from another brain region. We opted to use hippocampal tissue because it is a deeper brain structure that requires careful microdissection, frequently demanding extra time (Fig. 3A). Five-week-old mice were transcardially perfused with ice-cold 1 × PBS, and hippocampi were dissected out from both hemispheres at room temperature. We applied identical downstream steps for the hippocampal single-cell isolation as for the cerebral cortex. Animal preparation and micro-dissection of the hippocampi from one mouse required 15 min, thus the collection of the hippocampi from 3 mice was performed in 45 min. We obtained 5.2 × 10^5^ viable cells that were directly delivered for scRNA-seq processing and downstream analyses in a similar manner as for the cortices. After the quality control evaluation, we obtained ∼14.2 × 10^3^ cells, and all cell populations obtained in the cortex were also captured in the hippocampal tissue, with the addition of scarce of ependymal and epithelial cells (Fig. 3B and C). Captured cells were represented in all triplicates, and microglia were the most abundant cell type (∼40 %) followed by vascular cells and glial cells (both ∼20 %) (Fig. 3D and E).

These data indicate that our cell isolation method is resilient for the processing of time-demanding tissue dissections to obtain robust yields of microglia and vascular cells without a preselection step and is suitable for scRNA-seq.

### 3.4 Integrative across-time and across-region scRNA-seq analyses using cells obtained from distinct isolations

**Fig. 4:**
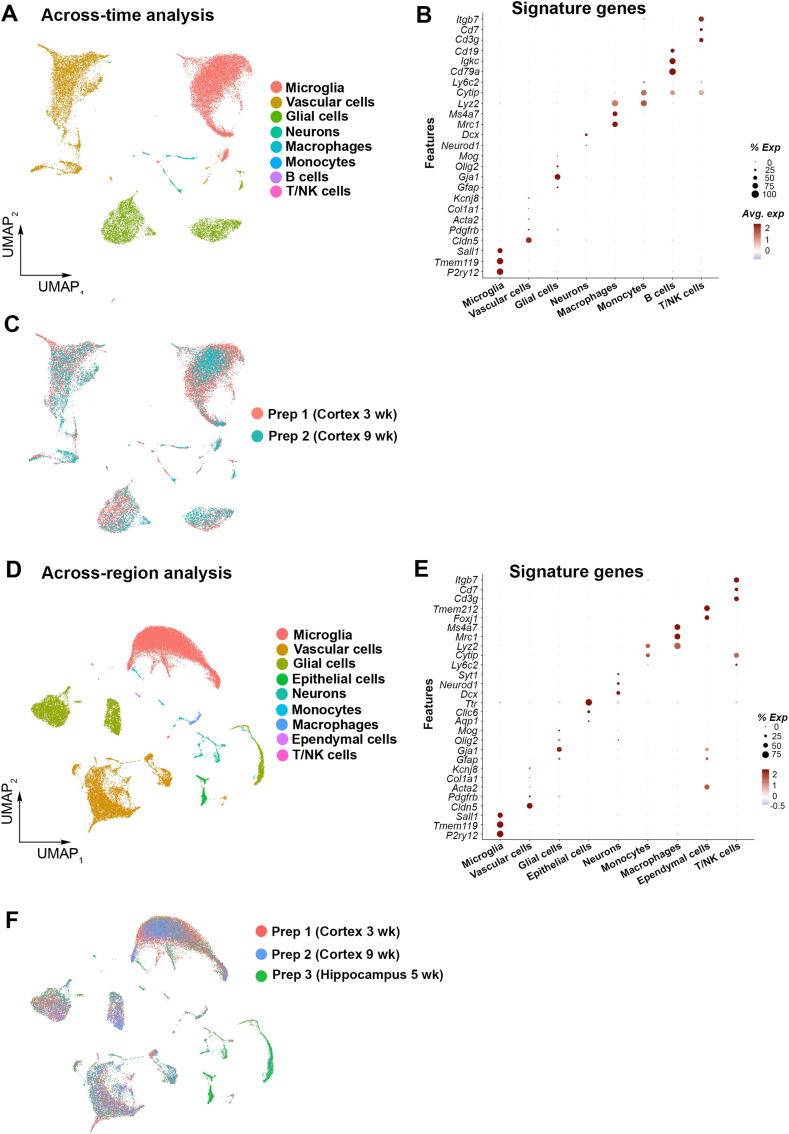
Integrative across-time and across-region scRNA-seq analyses using cells obtained from distinct isolations. (A) UMAP showing cell populations obtained when integrating scRNA-seq datasets from the two independent cortical preparations from three- and nine-week-old mice. (B) Dot plot showing the expression of signature genes identifying the captured cell populations (C) UMAP visualizing the cells obtained from each independent preparation. (D) UMAP showing cell populations obtained when integrating scRNA-seq datasets from the two cortical and hippocampal preparations. (E) Dot plot showing the expression of signature genes identifying the captured cell populations (F) UMAP visualizing the cells obtained from each preparation. Red = Prep 1: Cortex from three-week-old mice; Blue = Prep 2: Cortex from nine-week-old mice; Green = Prep 3: Hippocampus from five-week-old mice. (For interpretation of the references to colour in this figure legend, the reader is referred to the Web version of this article.)

To extend the versatility of our protocol, we next wanted to examine whether the scRNA-data generated using this isolation method would permit integration of cells obtained from independent preparations to perform across-time or across-region analyses. We were able to integrate the cells obtained from the two independent cortical preparations, three- and 9-week-old mice (across-time) (Fig. 4A and B). An overlap per cell type obtained from these independent preparations was observed, but the clustering pattern differed based on animal age (Fig. 4C). We were also able to integrate cells obtained from the cortical and the hippocampal preparations (across-region analysis) (Fig. 4D and E). An overlap per cell type from different regions was observed, except for those uniquely captured in the hippocampal samples (Fig. 4F). These data indicate that the concordance of scRNA-seq results generated from cells obtained with our isolation method would allow integration of multiple datasets collected over a different time course. This is valuable for implementing longitudinal molecular profiling studies on microglial and vascular cells within a brain region, for example during development or under a pathological condition.

## 4 Discussion

Longitudinal unbiased molecular profiling studies of the cellular components in distinct brain regions spanning multiple time points across physiological development or disease progression are largely lacking. Such studies would advance our understanding of the neurobiology of the brain under homeostasis and disease. This is even more important in systems undergoing frequent remodeling, such as immune responses and vasculature. Microglia are dynamic cells in nature and may adopt fluctuating molecular states during homeostasis while shaping the brain circuitry or during a temporal phase of pathological conditions [[12], [13], [14], [15]]. On the other hand, vascular remodeling is a hallmark of multiple CNS pathologies, such as stroke and neurodegeneration [16,17]. To date, there are no time series molecular studies on the responses of microglia or vascular cells at a single-cell level covering multiple time points. Besides the high cost of scRNA-seq sample processing, a technical challenge is the availability of reproducible methods allowing for robust, unbiased isolation of viable microglia or vascular cells from specific anatomical brain regions. Here, we describe a rapid and reproducible cell isolation method from perfused and micro-dissected brain tissue that produces particularly robust yields of microglia and vascular cells suitable for high throughput scRNA-seq. The reproducibility of this method would enable the creation of integrative longitudinal single-cell atlases for microglia and vascular cells for various purposes, such as under temporal courses of a disease progression or cellular responses across brain regions.

CNS tissue dissociation can be achieved by either mechanical homogenization, enzymatic digestion, or a combination of both. Mechanical homogenization allows rapid dissociation, has been shown to enable isolation of microglial cells from brain tissue [3,4], but has several limitations. It results in inconsistent numbers of viable cells and is ineffective in obtaining cells from structures rich in basal membranes, for example, when harvesting endothelial cells or stromal cells from blood vessels [18]. In this regard, enzymatic solutions are superior; however, the current isolation protocols prompt longer incubation durations, single or multi-step, at 37 °C that can exceed 1 h [2,19]. This longer *ex vivo* tissue handling at 37 °C raised concerns in transcriptomic profiling studies, as it influences the molecular states of the cells [4]. To overcome these limitations, we optimized our method to achieve an efficient tissue dissociation with only a single 10-min incubation in enzymatic solution to produce higher yields of microglia and vascular cells. The contents of the enzymatic solution and the duration of digestion are crucial for integrity of the cell outer structures, primarily surface and transmembrane receptors, which are vital for certain downstream applications, such as flow cytometry and cell sorting [6]. Using the enzyme mixture described in this method, we have succeeded in sorting microglia from the hippocampus using a fluorescence-activated cell sorting method based on expression of the transmembrane receptor CX3CR1 [20]. Thus, we anticipate that this cell isolation method will be valuable for recently emerging high-throughput proteogenomic analyses, such as cellular indexing of transcriptomes and epitopes (CITE-seq), where intact cell membrane epitopes are important [21]. As this technique is commonly used in immunological studies, and our cell isolation procedure is preceded by transcardial perfusion to eliminate blood and circulating immune cells and, furthermore, enriched for microglia, our cell isolation method is, hence, ideal to study neuroinflammation.

Myelin is an essential component of CNS tissue, found in fiber tracts around the neuronal axons to facilitate efficient nerve conduction [22]. Following CNS tissue dissociation, either through enzymatic digestion or mechanical homogenization, myelin degradation produces substantial amounts of debris. Myelin removal is, therefore, an essential step when a cell suspension will later be processed for an application relying on microfluidic diffusion. If this step is overlooked, clogging of the system may occur when performing flow cytometric analyses, cell sorting (both fluorescent or magnetic approaches), or scRNA-seq using the droplet-based method. In several microglial and endothelial cell isolation protocols, successful myelin removal was achieved through the use of a density gradient solution, often Percoll® [3,4], or myelin-depleting antibodies [2,5]. While these approaches demonstrate success in efficient myelin removal, this step demands longer processing times, ranging from 30 min [3] to over 1 h [2]. Here, we optimized our cell isolation method to achieve efficient myelin removal with a single Percoll® gradient that only required 10 min centrifugation, which also led to a significantly reduced *ex vivo* handling duration.

### 4.1 Limitations of the study

While enriching for microglia and vascular cells, our protocol also captures glial cells such as astrocytes and oligodendrocytes, offering the opportunity to study the molecular profiles of these cell types in a single preparation. Nevertheless, this isolation method fails to consistently capture neurons. Our ongoing work focuses on refining the method to collect all CNS cell types, including neurons, from micro-dissected brain regions in a simple and rapid preparation. It is worth noting that after the last step of generating the single-cell suspension, we pooled the cells obtained from three animals before processing for scRNA-seq, as the purpose of the current study was to demonstrate the technical feasibility of the method. In studies intended for comparing experimental conditions, pooling cells from serval animals might not be optimal, and thus processing of the cells obtained from each individual animal separately for scRNA-seq would be ideal. If pooling the cells is necessary, a cell tagging approach to identify the cells originating from each animal would be an option [23]. Additionally, the current study was performed using juvenile and young adult mice. In studies performed in older mice, the outcome of the method may differ as the amount of myelin debris is expected to be higher in aged mice. In this case, the myelin removal step may need modification. Finally, previous reports have claimed that longer incubations with enzymatic solutions containing high amounts of dispase (such as 5 mg/ml) may affect certain surface antigens, especially in leukocytes [24,25]. The enzymatic mixture used in our method contains a lower amount of dispase II (1 mg/ml), and the tissue is exposed to the enzyme for only 10 min. Although we previously succeeded in isolating microglia based on a transmembrane receptor, this concern should be taken into consideration as the integrity of membrane proteins when exposed to dispase may vary depending on the target protein. Moreover, as the principal objective of our method was to avoid bias during population selection, we did not want to introduce an extra step potentially skewing the proportions of microglia and vascular cells obtained after a flow cytometric sorting. Skewing the proportions of these cell types can occur at the initial steps of flow cytometry, for example, when selecting the cells based on their cell size (forward scatter) or granularity (side scatter); especially under pathological conditions when microglia become reactive and adopt phagocytic states. Importantly, the proportional representations of microglia and vascular cells were conserved in the single-cell analyses performed on the cell suspensions generated using our isolation obtained from three independent preparations from mouse brain tissues collected at different ages and subregions.

## Data availability

The accession numbers for the single-cell RNA-seq data reported in this paper are GEO: GSE270158 for cerebral cortex of three-week-old mice and the hippocampus of five-week-old mice; and GSE197360 for cerebral cortex of nine-week-old mice.

## CRediT authorship contribution statement

**Efthalia Preka:** Writing – review & editing, Writing – original draft, Visualization, Validation, Methodology, Investigation, Formal analysis, Data curation. **Alejandro Lastra Romero:** Writing – review & editing, Writing – original draft, Methodology, Data curation. **Ying Sun:** Writing – review & editing, Visualization, Validation, Methodology, Data curation. **Yara Onetti Vilalta:** Writing – review & editing, Methodology. **Thea Seitz:** Writing – review & editing, Writing – original draft. **Adamantia Fragkopoulou:** Writing – review & editing, Writing – original draft, Supervision. **Christer Betsholtz:** Writing – review & editing, Supervision. **Ahmed M. Osman:** Writing – review & editing, Writing – original draft, Supervision, Methodology, Investigation, Conceptualization. **Klas Blomgren:** Writing – review & editing, Writing – original draft, Supervision, Project administration, Methodology, Investigation, Funding acquisition, Conceptualization.

## Declaration of competing interest

The authors declare that they have no known competing financial interests or personal relationships that could have appeared to influence the work reported in this paper.
