# Supplementary figures and images for: Rapid and robust isolation of microglia and vascular cells from brain subregions for integrative single-cell analyses
Source: Heliyon. 2024 Aug 5;10(16):e35838. doi: 10.1016/j.heliyon.2024.e35838 (PMC11357767; doi:10.1016/j.heliyon.2024.e35838)

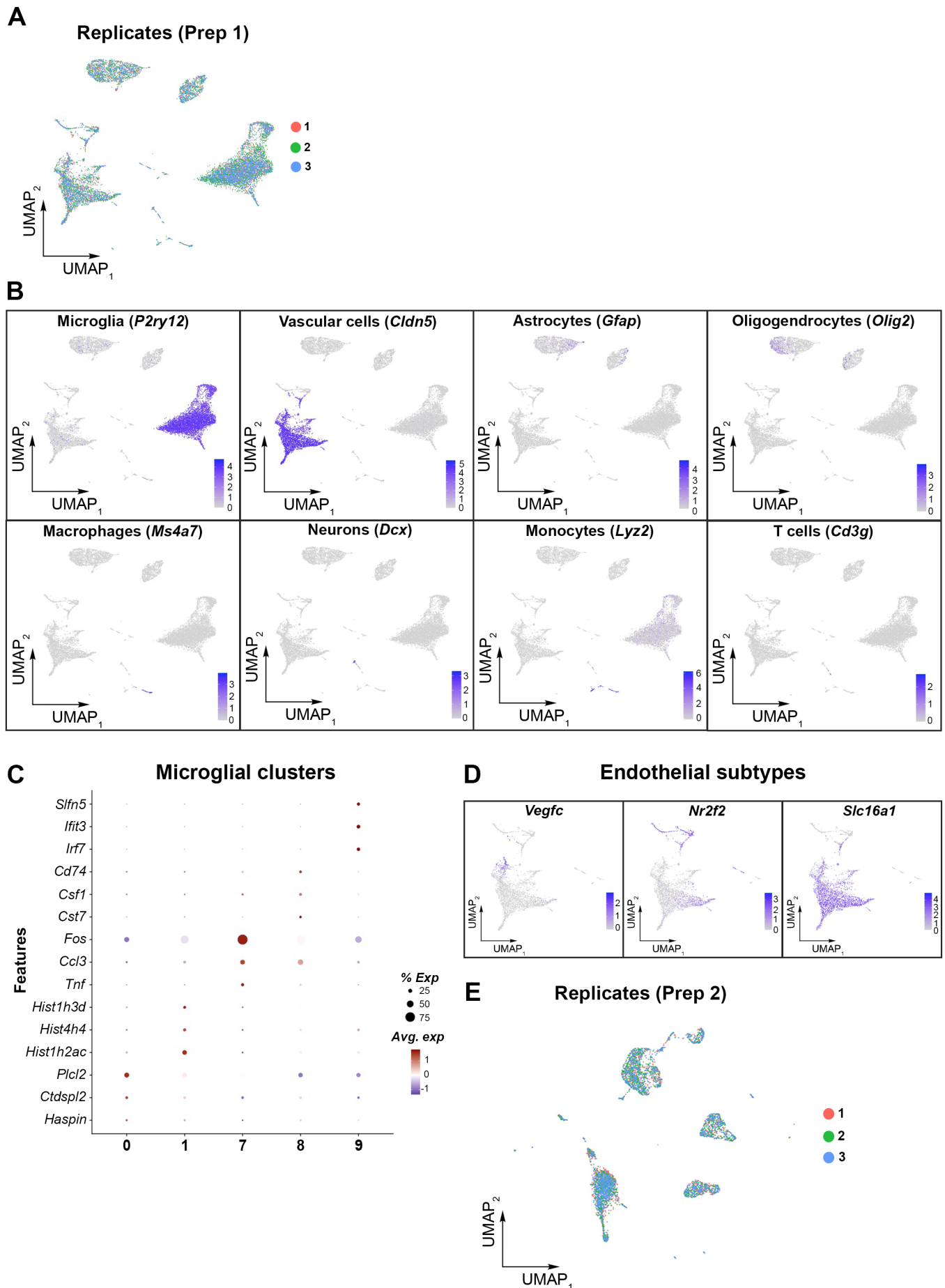

Supplementary figure 1

Supplement: Multimedia component 1 [file mmc1.pdf]
